# Subcellular Epithelial HMGB1 Expression Is Associated with Colorectal Neoplastic Progression, Male Sex, Mismatch Repair Protein Expression, Lymph Node Positivity, and an ‘Immune Cold’ Phenotype Associated with Poor Survival

**DOI:** 10.3390/cancers15061865

**Published:** 2023-03-20

**Authors:** Ross J. Porter, Graeme I. Murray, Sandra Hapca, Andrew Hay, Stephanie G. Craig, Matthew P. Humphries, Jacqueline A. James, Manuel Salto-Tellez, Daniel P. Brice, Susan H. Berry, Mairi H. McLean

**Affiliations:** 1 Centre for Inflammation Research, Queen’s Medical Research Institute, University of Edinburgh, Edinburgh EH16 4TJ, UK; 2 Institute of Medical Sciences, School of Medicine, Medical Sciences & Nutrition, University of Aberdeen, Aberdeen AB25 2ZD, UK; 3 Precision Medicine Centre of Excellence, Centre for Cell Research and Cell Biology, Queen’s University Belfast, Belfast BT9 7BL, UK; 4 Department of Cellular Pathology, Royal Victoria Hospital, Belfast Health and Social Care Trust, Belfast BT12 6BA, UK; 5 Lydia Becker Institute of Immunology and Inflammation and Wellcome Centre for Cell-Matrix Research, University of Manchester, Manchester M13 9WU, UK; 6 Division of Molecular & Clinical Medicine, School of Medicine, University of Dundee, Dundee DD1 9SY, UK

**Keywords:** HMGB1, colorectal cancer, mismatch repair, lymphocytes, cytokine, therapy

## Abstract

**Simple Summary:**

New treatment targets are urgently needed for colorectal cancer. Here, we investigate the role of HMGB1—a multifunctional immune protein—in colorectal cancer. We demonstrate dynamic subcellular (nuclear and cytoplasmic) HMGB1 expression in lesions seen throughout different stages of colorectal cancer development. In cancer, HMGB1 is linked, for the first time, to tumour progression, lymph node metastases, male sex, and key biological parameters of mismatch repair protein expression and stromal immune cell phenotype. Strong cytoplasmic HMGB1 expression is also associated with an ‘immune cold’ tumour microenvironment, which is associated with poor survival. HMGB1 may therefore represent a novel treatment target for colorectal cancer.

**Abstract:**

New treatment targets are needed for colorectal cancer (CRC). We define expression of High Mobility Group Box 1 (HMGB1) protein throughout colorectal neoplastic progression and examine the biological consequences of aberrant expression. HMGB1 is a ubiquitously expressed nuclear protein that shuttles to the cytoplasm under cellular stress. HMGB1 impacts cellular responses, acting as a cytokine when secreted. A total of 846 human tissue samples were retrieved; 6242 immunohistochemically stained sections were reviewed. Subcellular epithelial HMGB1 expression was assessed in a CRC Tissue Microarray (*n* = 650), normal colonic epithelium (*n* = 75), adenomatous polyps (*n* = 52), and CRC polyps (CaP, *n* = 69). Stromal lymphocyte phenotype was assessed in the CRC microarray and a subgroup of CaP. Normal colonic epithelium has strong nuclear and absent cytoplasmic HMGB1. With progression to CRC, there is an emergence of strong cytoplasmic HMGB1 (*p* < 0.001), pronounced at the leading cancer edge within CaP (*p* < 0.001), and reduction in nuclear HMGB1 (*p* < 0.001). In CRC, absent nuclear HMGB1 is associated with mismatch repair proteins (*p* = 0.001). Stronger cytoplasmic HMGB1 is associated with lymph node positivity (*p* < 0.001) and male sex (*p* = 0.009). Stronger nuclear (*p* = 0.011) and cytoplasmic (*p* = 0.002) HMGB1 is associated with greater CD4^+^ T-cell density, stronger nuclear HMGB1 is associated with greater FOXP3^+^ (*p* < 0.001) and ICOS^+^ (*p* = 0.018) lymphocyte density, and stronger nuclear HMGB1 is associated with reduced CD8^+^ T-cell density (*p* = 0.022). HMGB1 does not directly impact survival but is associated with an ‘immune cold’ tumour microenvironment which is associated with poor survival (*p* < 0.001). HMGB1 may represent a new treatment target for CRC.

## 1. Introduction

Colorectal cancer (CRC) is one of the most common malignancies worldwide, representing one in ten cancer cases and deaths [1]. Despite advances in treatments and earlier detection through national screening programs, patient mortality remains high. Therefore, there is a clinical need to increase understanding of the pathogenesis of this malignancy and to develop prognostic biomarkers and new treatment targets. The adenoma-carcinoma hypothesis, whereby normal columnar colorectal epithelium transforms into adenoma and eventually invasive carcinoma, has been the most widely accepted model of sporadic tumourigenesis [2,3]. Knowledge of the genetic and epigenetic events associated with this malignancy continue to evolve, leading to different molecular classifications (termed consensus molecular subtypes, identified through bulk transcriptomic analysis) to predict prognosis and response to treatments [4,5].

Inflammation is a critical hallmark of cancer and, depending upon the immune cell populations involved, stage of disease and cancer type, can be either protective or pathogenic [5,6]. In the colon, inflammation drives cancer progression. However, distinct immune cell populations such as CD8^+^ T-cells have demonstrated potent anti-tumour activity [7]. A better understanding of critical regulators of cancer-associated inflammation is essential for development of novel therapeutic strategies for CRC.

With this in mind, our aim was to characterise the role of the protein High Mobility Group Box 1 (HMGB1) in colorectal neoplastic progression. HMGB1 is a multi-functional, ubiquitous, highly conserved protein expressed by most epithelial and immune cells [8]. Under normal physiological conditions, HMGB1 localises to the nucleus where it binds to the minor groove of DNA, without sequence specificity, to stabilise the genome, regulate transcription and enhance DNA repair processes [8,9]. HMGB1 can undergo post-translational modification such as acetylation, methylation and phosphorylation, resulting in cytoplasmic shuttling and subsequent extracellular release. HMGB1 can reach the extra-cellular space through active secretion or passive release from damaged or dying cells to act as a damage-associated molecular pattern molecule [8,9]. Within the inflammatory milieu, HMGB1 shares many properties with other damage associated molecular patterns, potentiating inflammation by influencing epithelial and immune cell responses involved in cell proliferation, cell migration, tissue regeneration, wound healing and vessel remodelling [10,11]. Therefore, HMGB1 has the potential to impact on neoplastic progression across both epithelial cell and immune cell driven pathways.

HMGB1 expression has been associated with almost all epithelial-derived malignancies, where it can promote both pro-tumour and anti-tumour responses [12]. The expression and biological significance of HMGB1 in colorectal tumorigenesis is not understood, and therefore warrants investigation. This study defines the dynamic subcellular epithelial expression of HMGB1 throughout colorectal neoplastic progression and investigates downstream biological consequences of aberrant expression. We demonstrate that HMGB1 expression is associated with colorectal neoplastic progression, male sex, mismatch repair protein expression, lymph node metastases and changes to stromal immune cell infiltration in CRC.

## 2. Materials and Methods

### 2.1. Tissue Specimens

A total of 6242 immunostained sections from 846 human colonic tissue samples were used across a colorectal cancer (CRC) tissue microarray (TMA) and archival formalin-fixed paraffin-embedded (FFPE) biopsy and endoscopic polypectomy specimens.

### 2.2. Colorectal Cancer Tissue Microarray

FFPE tissue cores from 650 CRCs and 50 matched normal pairs were presented within a previously validated TMA [13,14,15]. Tissue cores were obtained from chemotherapy and radiotherapy-naïve patients undergoing elective surgery for primary CRC between 1994 and 2009 at Aberdeen Royal Infirmary, Scotland UK. Clinico-pathological data, including survival up to 18.2 years, were available for each case as described in Supplementary File S1.

### 2.3. Colonic Tissue Biopsies and Endoscopic Polypectomy Specimens

A total of 52 colorectal adenomatous polyps, 69 colorectal cancer polyps (CaP) (28 initial cohort plus 41 validation cohort), and 25 normal colonic mucosal biopsies were retrieved from the respective number of patients at time of colonoscopy or surgery at Aberdeen Royal Infirmary. Tissues was collected between 2010–2015 for all except the CaP validation cohort that was collected in 2019. Sections of whole CaP lesions were used for assessment: all CaP lesions had distinct foci of carcinoma on a background of adenoma, and 20 (90.9%) had foci of normal epithelium at their base (initial cohort). Full characterisation of all polyp tissue specimens is described in Supplementary File S2. Histological diagnosis was confirmed in all samples by an expert gastrointestinal pathologist (GIM). Tissue was selected to represent colorectal neoplastic progression and sections were excluded if they were not representative of pathology. Anonymised and matched molecular data were available through the NHS Grampian Biorepository and Pathology Database for the validation CaP cohort.

### 2.4. Immunohistochemistry

Intensity and subcellular localisation (nuclear +/− cytoplasmic) of epithelial HMGB1 was assessed immunohistochemically in all FFPE tissue specimens (*n* = 846). Epithelial expression of p53 and RUNX3, and stromal inflammatory cell phenotype were also assessed at the invasive cancer margin in CaP (*n* = 28 initial cohort). Immune cell phenotyping was performed on serial sections of these CaP lesions where CD68, CD20, CD4, CD8 and FOXP3 identified macrophages, B-cells, and helper, cytotoxic and regulatory (Treg) T-cell subsets, respectively. These epithelial and immune cell markers were chosen because of previously established biology between them and HMGB1 or cancer [13,16]. Immunohistochemistry was performed using a Dako Autostainer (Dako, Ely, UK), as previously described [14,15,16]. Antigen retrieval was performed in 10 mM citrate (pH6) or EDTA (pH 7.8) buffer for 20 min, primary antibody was applied for 60 min, and EnVision+^™^ peroxidase-linked biotin-free synthesis (Dako, Ely, UK) with 3′-3′-diaminobenzidine as chromogen was used for detection. Optimised methodologies and antibody specifications are detailed in Supplementary File S3.

The immunohistochemical assessment of lymphocyte infiltrate (CD3^+^, CD4^+^, CD8^+^, FOXP3^+^, CD20^+^ cells) and immune checkpoint biomarkers (IDO-1, ICOS, PDL1) on the CRC TMA was performed as part of a different study as previously published [13], and the raw cell density data were obtained through the Grampian Biorepository for secondary analysis. Details of the antibodies and staining conditions are summarised in Supplementary File S3.

### 2.5. Scoring of Immunohistochemistry

Stained sections for HMGB1 expression in the TMA, and HMGB1, p53, RUNX3, CD4, CD8, CD20, FOXP3, CD20, and CD68 expression in polyp lesions were independently reviewed under light microscopy by two observers. For assessment of epithelial HMGB1, p53 and RUNX3 expression, a previously published, semi-quantitative scoring methodology was used [14,15,16]. In brief, intensity was classified as absent, weak, moderate or strong. Specimens with discordant scores were re-assessed by both observers simultaneously to reach consensual agreement.

For assessment of the immune cell infiltrate in CaP, the invasive cell margin was identified in each lesion by an expert gastrointestinal pathologist (GIM) in serial sections and the immediately adjacent immune cell infiltrate was captured by digital imaging and quantified as number of positive cells per high power field (×20 magnification), as previously published [17]

For immune markers on the CRC TMA, stained slides were scanned using a Leica Aperio AT2 whole slide scanner at ×40 magnification. Digital image analysis was then performed using QuPath software version 0.1.2. [18], as previously published [13]. In summary, a senior consultant pathologist assisted in annotating the cores before an automated scoring algorithm was applied for each biomarker. An average density of positive cells present for all TMA cores available for each patient was then calculated. Cases were excluded if interpretation of the immunostaining was not possible, for example due to folded or damaged cores. For this study, continuous cell counts were used. In addition, immune cell data were categorised into ‘immune cold’ (defined as either low CD3, CD4 and CD8, or low CD3 and CD8 densities) versus immune not otherwise specified (NOS) phenotypes, given the association between an ‘immune cold’ phenotype and poorer survival identified by Craig et al., within the TMA [13]

### 2.6. Data Analysis and Statistics

Analysis for immunohistochemistry was carried out using Pearson’s chi square test, Fisher’s exact test, and log-rank test with Kaplan–Meier survival analysis, as indicated. For association between epithelial HMGB1 expression and the immune microenvironment, Mann–Whitney U and Kruskal–Wallis tests were used for comparisons when there were two or greater than two comparator groups, respectively. IBM^®^SPSS^®^ Version 25.0 (IBM, Portsmouth, UK) or Prism Version 9 (GraphPad Software Inc., San Diego, CA, USA) was used. A two-tailed alpha was set at 0.05. For immunohistochemistry experiments, semi-quantitative scores for HMGB1, p53 and RUNX3 expression were also dichotomised using negative vs. positive staining, negative and weak staining vs. moderate and strong staining, and strong vs. negative/weak/moderate staining comparisons as previously published [14,15,16]. Analysis of the immune cell infiltrate in the CRC TMA used continuous density counts determined via QuPath [18], as previously published [13]

## 3. Results

**Figure 1:**
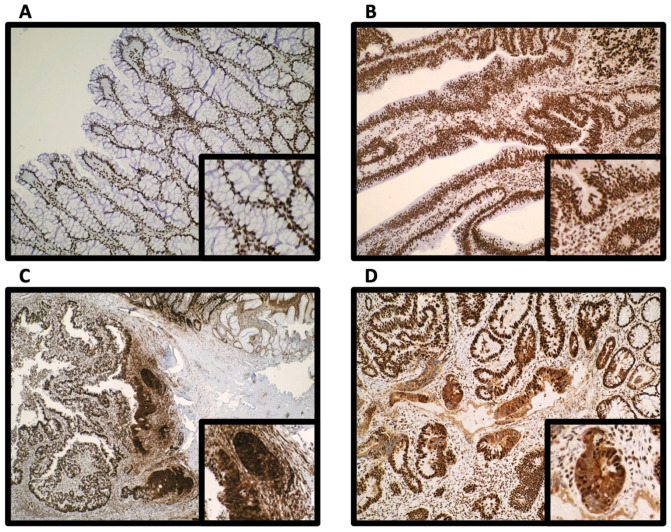
Representative photomicrographs. (**A**) Strong nuclear and absent cytoplasmic HMGB1 expression in normal colonic epithelium; (**B**) Strong nuclear and weak cytoplasmic HMGB1 in benign adenomatous colorectal polyps; (**C**) Strong nuclear and cytoplasmic HMGB1 expression at the invasive cancer margin within a colorectal cancer polyp and (**D**) Strong nuclear and cytoplasmic HMGB1 expression in colorectal cancer. Photomicrographs were taken by expert GI pathologist (GIM) with a digital Olympus camera (Olympus, Tokyo, Japan), ×20 magnification. Right corner box represents higher magnification images.

Representative photomicrographs of epithelial nuclear and cytoplasmic HMGB1 expression intensity across colorectal neoplastic progression are shown in Figure 1 and Supplementary File S4.

### 3.1. Subcellular Epithelial HMGB1 Expression Is Associated with Colonic Cancer, Lymph Node Positivity and Mismatch Repair Protein Expression

**Figure 2:**
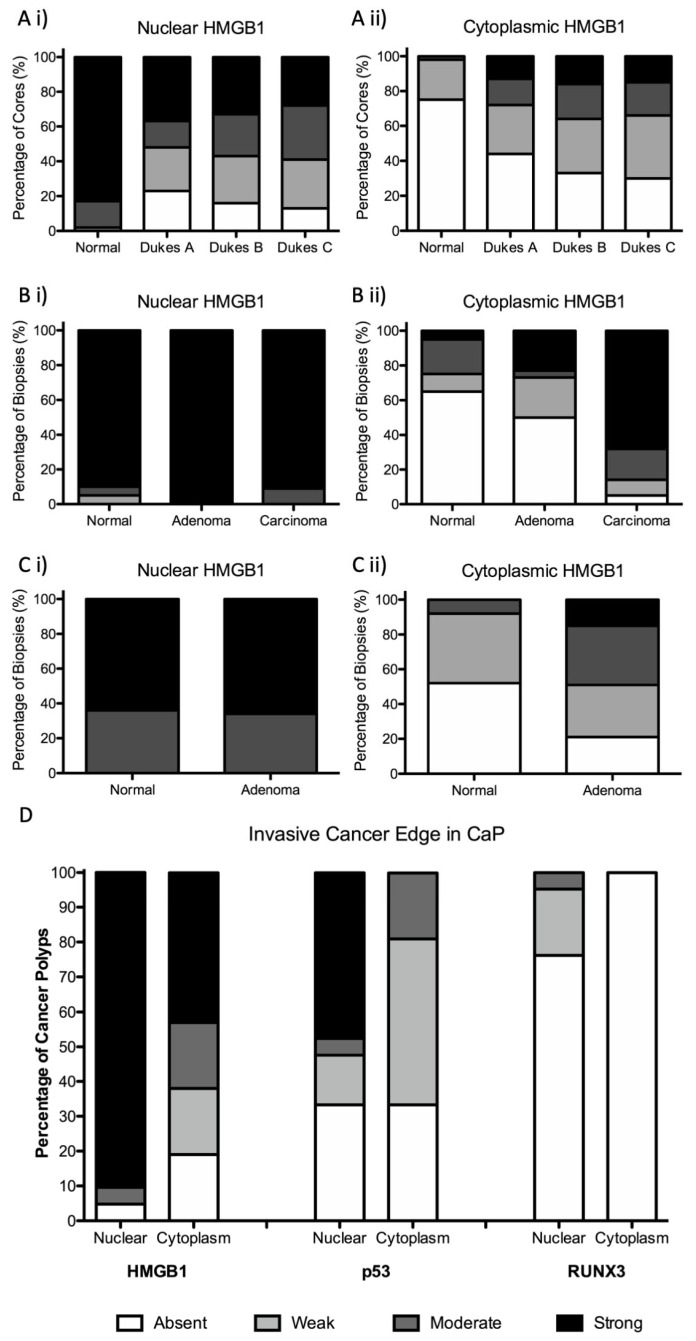
Emergence of cytoplasmic HMGB1 and reduction in nuclear HMGB1 is associated with the adenoma–carcinoma sequence. Intensity of (i) epithelial nuclear and (ii) epithelial cytoplasmic HMGB1 expression in (**A**) colorectal cancer; (**B**) colorectal cancer polyps; and (**C**) benign adenomatous polyps. (**D**) Epithelial nuclear and cytoplasmic expression of HMGB1, p53 and RUNX3 at the invasive cancer margin in cancer polyps (CaP).

We characterised subcellular epithelial cell expression of HMGB1 in the colorectal cancer (CRC) TMA (*n* = 650) (Table 1). HMGB1 was strongly expressed in the nucleus of normal colonic epithelial cells, and this was reduced in colon cancer (*p* < 0.001). The switch to reduced nuclear HMGB1 expression was between normal and T1 tumour stage (*p* < 0.001). Thereafter, increased intensity of nuclear expression was associated with increasing TNM (*p* = 0.008) and Dukes’ (*p* = 0.016) stage (Figure 2A, Supplementary File S5). Cytoplasmic HMGB1 was not expressed by most normal colonic epithelial cells. Conversely, increased cytoplasmic expression emerged in colon cancer (*p* < 0.001). Overall, we reveal that reduction of nuclear and emergence in cytoplasmic epithelial HMGB1 occurs in colorectal cancer.

We then considered if this dynamic HMGB1 expression profile was associated with clinical (such as age, sex, tumour site) and pathological (such as differentiation, EMVI, mismatch repair protein expression) parameters. The presence of cytoplasmic HMGB1 was associated with lymph node positivity (*p* < 0.001). Absent (*p* = 0.001) or weak (*p* = 0.010) nuclear HMGB1 expression was associated with mismatch repair protein expression, specifically loss of MLH1 and MSH2 protein expression. Males were more likely to express stronger cytoplasmic HMGB1 (*p* = 0.009) (Supplementary File S5). There was no direct relationship between nuclear (*p* = 0.213) or cytoplasmic (*p* = 0.498) HMGB1 expression and overall survival (Supplementary File S6).

### 3.2. Epithelial Cytoplasmic HMGB1 Is Increased in Endoscopically Resected Colonic Adenomas

As reduction of nuclear HMGB1 and emergence in cytoplasmic HMGB1 expression was identified in established CRC, we investigated the expression pattern of HMGB1 throughout the adenoma–carcinoma sequence. First, we assessed expression in benign endoscopically resected adenomatous polyps compared with adjacent normal mucosa. While there was no difference in nuclear HMGB1 expression intensity, benign colorectal adenomatous polyps expressed stronger epithelial cytoplastic HMGB1 compared with normal colonic epithelium (*p* = 0.004) (Table 1 and Figure 2C).

### 3.3. Epithelial HMGB1 Expression Is Prominent at the Leading Edge of Polyp Cancers (CaP)

We next assessed expression of HMGB1 in polyps displaying a focus of cancer (termed cancer polyps, CaP). CaP represent an increasing proportion of CRC diagnosed through the national screening program, and some can progress such as to lymph node metastases [19]. These lesions offer an opportunity to assess protein expression across normal, adenoma and cancer in each single lesion. In CaP, there was no difference between intensity of nuclear HMGB1 expression in areas of carcinoma compared with adjacent normal or adenomatous epithelium. However, areas of carcinoma expressed significantly stronger intensity of cytoplasmic HMGB1 compared with adjacent normal epithelium (*p* < 0.001) and adenoma (*p* < 0.002) (Table 1, Figure 2B and Supplementary File S7).

**Figure 3:**
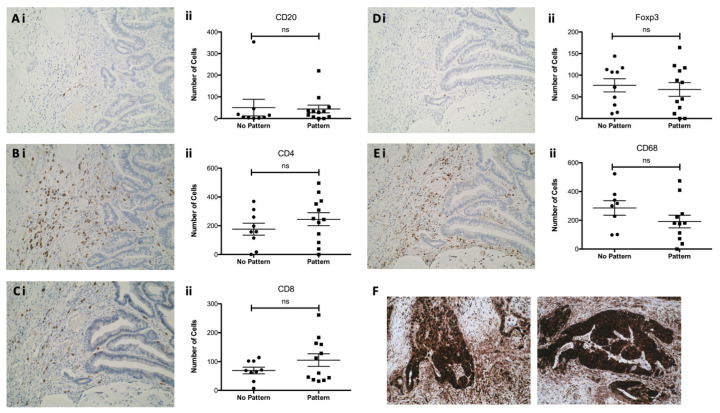
Immune cell infiltrate at the invasive cancer margin in cancer polyps. There is a preponderance of CD4^+^ T-cells and CD68^+^ macrophages. Immune cell phenotype is not associated with HMGB1 expression pattern (characterised as strong nuclear and cytoplasmic expression). Panels represent (i) representative photomicrographs and (ii) lymphocyte association with HMGB1 expression signature at the invasive cancer margin for (**A**) CD20^+^ B-cells; (**B**) CD4^+^ T-cells; (**C**) CD8^+^ T-cells; (**D**) FOXP3^+^ Tregs; and (**E**) CD68^+^ macrophages. (**F**) represents strong nuclear and cytoplasmic HMGB1 expression pattern at the invasive cancer margin in two cancer polyp lesions. Photomicrographs were taken by expert GI pathologist (GIM) with a digital Olympus camera (Olympus, Tokyo, Japan), ×20 magnification. ns = not significant.

While assessing HMGB1 expression in CaP, there was a striking pattern of moderate/strong nuclear and cytoplasmic HMGB1 expression apparent in 77% of the CaP at the invasive cancer margin, the transitional space where cancerous cells invade normal or adenomatous epithelium (Figure 1 and Figure 3F). We validated and confirmed this CaP expression profile in a second CaP cohort (*n* = 41). In this cohort, 82.1% of CaP expressed the pattern of moderate/strong nuclear and cytoplasmic HMGB1.

### 3.4. Epithelial HMGB1 Is Not Associated with Epithelial p53 or RUNX3 Expression at the Invasive Cancer Margin or Molecular Phenotype

To further investigate the consequences of HMGB1 expression on epithelial cell responses at the invasive cancer margin in CaP, we assessed the relationship between HMGB1 and expression of downstream effector proteins p53 and RUNX3 in the original CaP cohort. Nuclear and cytoplasmic HMGB1, p53 and RUNX3 were differentially expressed at the CaP invasive cancer margin. However, there was no relationship between HMGB1 and p53 or HMGB1 and RUNX3 expression patterns (Figure 2D). There was no association between HMGB1 (either nuclear, cytoplasmic or strong nuclear + cytoplasmic expression together) and *KRAS*, *BRAF*, or microsatellite instability (MSI) status.

### 3.5. Epithelial HMGB1 at the Invasive Edge in CaP Is Not Associated with a Differential Adjacent Immune Cell Phenotype

We continued to investigate the biological significance of HMGB1 expression profile on the microenvironment at the invasive cancer margin within CaP lesions, by defining the inflammatory cell phenotype in this area. CD4^+^ T-cells and CD68^+^ macrophages predominate in the stroma around the invasive cancer margin in these lesions. CaP displaying the strong cytoplasmic and nuclear HMGB1 signature pattern did not display a change in adjacent stromal immune cell phenotype, compared to those CaP that did not display this invasive edge HMGB1 signature pattern, as outlined in Figure 3 and Supplementary File S8.

### 3.6. Epithelial HMGB1 Expression Is Associated with Increased Stromal CD4^+^, FOXP3^+^ and ICOS^+^ Cells, and Reduced CD8^+^ Cells in Colorectal Cancer

**Figure 4:**
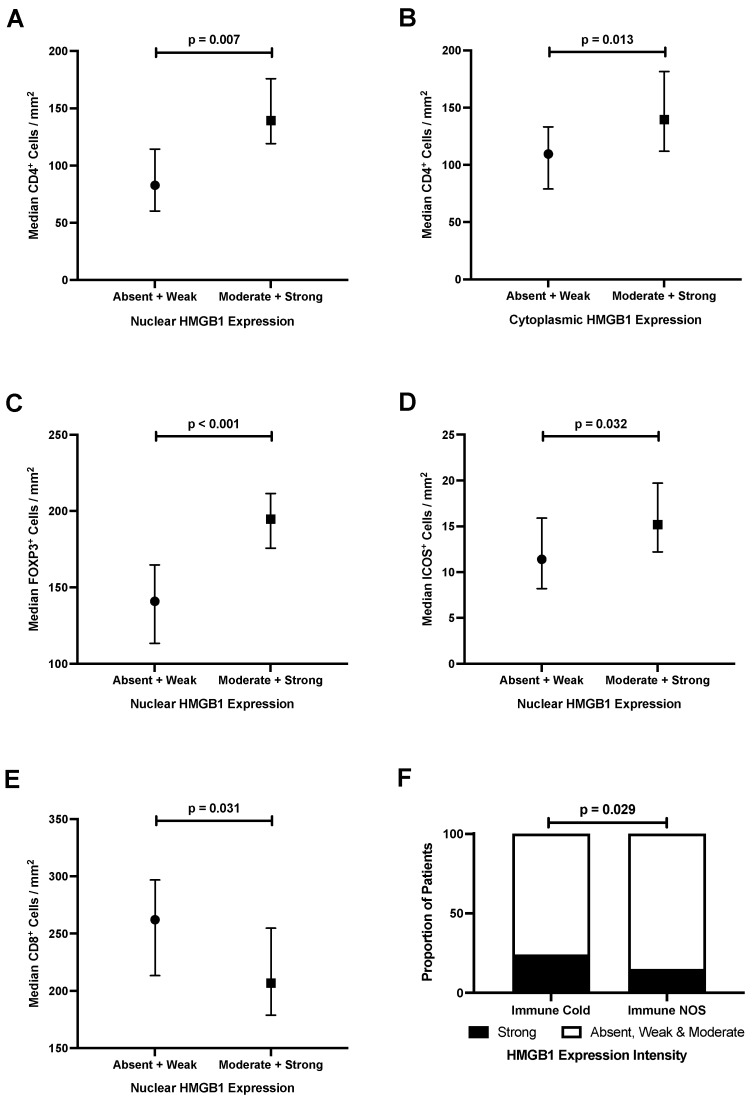
HMGB1 expression is associated with the immune cell infiltrate in colorectal cancer. (**A**) stronger nuclear HMGB1 expression is associated with a greater density of CD4^+^ lymphocytes; (**B**) stronger cytoplasmic HMGB1 expression is associated with a greater density of CD4^+^ lymphocytes; (**C**) stronger nuclear HMGB1 expression is associated with a greater density of FOXP3^+^ lymphocytes; (**D**) stronger nuclear HMGB1 expression is associated with a greater density of ICOS^+^ lymphocytes; (**E**) stronger nuclear HMGB1 expression is associated with a lower density of CD8^+^ lymphocytes; (**F**) strong cytoplasmic HMGB1 is associated with ‘immune cold’ colorectal cancers. Data analysed by Man–Whitney U or Chi square tests and expressed as median cell density (95% CI error bars).

The stromal immune cell response is prognostic for CRC and measured clinically by the consensus immunoscore as a prognostic biomarker to aid treatment planning [20,21]. As HMGB1 can act as a damage associated molecular pattern in the extracellular space to modulate immune responses, we next focused our attention back to the CRC TMA (*n* = 650, Supplementary File S1) to assess whether epithelial HMGB1 expression was associated with the tumour immune microenvironment (Table 2 and Figure 4). Strong nuclear (*p* = 0.011) and cytoplasmic (*p* = 0.002) HMGB1 expression was associated with an increased density of CD4^+^ T-cells. Strong nuclear HMGB1 expression was also associated with increased density of FOXP3^+^ immune cells (*p* < 0.001) and increased density of ICOS^+^ lymphocytes (*p* = 0.018). The presence of nuclear HMGB1 was associated with reduced density of CD8^+^ T-cells (*p* = 0.022). There was no association between HMGB1 expression and CD20^+^ B-cell immune infiltrate in CRC.

### 3.7. Strong Epithelial Cytoplasmic HMGB1 Is Associated with Immune Checkpoint Markers and an ‘Immune Cold’ Phenotype That Confers Poor Prognosis in Colorectal Cancer

‘Immune cold’ tumours (determined by low density of CD3^+^, CD4^+^ and CD8^+^ cells) were the best prognosticator for CRCs within the TMA, as reported by Craig et al. [13]. This was confirmed using raw data for secondary analysis, which reported that patients with ‘immune cold’ tumours had poorer survival compared with patients with ‘immune NOS’ tumours (*p* < 0.001) (Supplementary File S9).

Further, we report that strong cytoplasmic HMGB1 was associated with ‘immune cold’ tumours (24% versus 15% for immune cold and NOS, respectively, *p* = 0.029) (Table 2 and Figure 4). Therefore, while epithelial HMGB1 doesn’t directly impact survival, strong cytoplasmic HMGB1 expression is associated with an ‘immune cold’ tumour microenvironment which we have demonstrated confers a poor prognosis in CRC. While numbers are relatively small and the underlying mechanism remains unclear, strong epithelial cytoplasmic HMGB1 expression is also associated with lower expression of immune checkpoint markers IDO-1 (*p* = 0.010), ICOS (*p* = 0.002), and stromal PDL1 (*p* = 0.027) (Table 2).

## 4. Discussion

We have defined the expression of HMGB1 in human colorectal neoplastic progression from normal mucosa through pre-malignant adenomatous polyps, cancer polyps, and CRC of increasing stage. In our large, well characterised cohort, we have identified an HMGB1 expression profile switch with dynamic subcellular localisation between normal and T1 malignancy, and a striking HMGB1 expression signature at the leading invasive edge in the majority of polyp cancer lesions. In established CRC, this subcellular HMGB1 expression profile is linked to tumour progression and, for the first time, lymph node metastases and key biological parameters of mismatch repair protein expression, male sex, and stromal immunophenotype. While HMGB1 expression is not directly associated with survival, strong epithelial cytoplasmic HMGB1 is associated with an ‘immune cold’ tumour microenvironment which is associated with poor survival outcomes. These data reveal novel insight into both HMGB1 biology and colorectal neoplastic progression and suggest that HMGB1 should be explored as a novel therapeutic target for CRC.

Dynamic subcellular expression of HMGB1 has been reported previously in a few smaller studies of CRC [12,22,23]. Wang and colleagues demonstrated positive nuclear expression with no cytoplasmic HMGB1 in normal colonic epithelium, as we do here, and emergence of cytoplasmic HMGB1 in their smaller cohort of adenomas (*n* = 68) and colorectal cancers (*n* = 369) [24]. In their study, there was a significant difference in survival based on HMGB1 expression pattern. This impact on survival was also identified in another small cohort of 72 cases of colonic cancer [22]. Both studies were based in China and differences in survival may reflect geographical variations in environmental exposures resulting in different molecular drivers of disease. From our data, HMGB1 does not appear to be a direct prognostic biomarker for survival.

A key question is whether the emergence of cytoplasmic HMGB1 is protective or pathogenic, particularly at the leading edge of cancer polyps (CaP). The use of polyp cancer lesions to explore this question offers the unique opportunity to assess HMGB1 expression in normal, adenomatous and malignant epithelium within the same lesion, eliminating inter-patient confounding. We first explored expression of downstream effector proteins p53 and RUNX3 at the invasive cancer margin of CaP, where the most striking pattern of strong nuclear and cytoplasmic expression of HMGB1 was seen in the majority of lesions. *p53* is a tumour suppressor gene associated with CRC [25]. HMGB1 can facilitate p53-DNA binding, induce a p53-dependent senescent growth arrest, and form complexes with p53 to mediate autophagy and apoptosis [26,27]. RUNX3 is a transcription factor implicated in lymphocyte immune responses and linked to HMGB1 [28]. Our data did not demonstrate a relationship between HMGB1 expression and p53 or RUNX3 at the invasive cancer margin. Nor did we find an association between HMGB1 and the molecular phenotype of these CaP lesions, investigating *BRAF*, *KRAS* and MSI status chosen to reflect the molecular phenotyping used in clinical practice to direct treatment options in established disease.

In addition to the emergence of cytoplasmic HMGB1, we also report reduced nuclear HMGB1 expression in early stage (pT1) CRC compared with normal epithelium. There are little published data regarding the consequences of reduced nuclear HMGB1 expression. One consequence of nuclear HMGB1 depletion may be induction of cellular senescence, the cellular response mechanism whereby proliferation is arrested in response to a potentially carcinogenic insult, as this mechanism has been reported in human mammary epithelial cells in vitro [29]. However, there may be alternative or additional explanations. For example, nuclear HMGB1 binds to the minor groove of DNA, without sequence specificity, to provide structural support, stabilise the genome, regulate transcription, and enhance DNA repair processes [30]. Reduced nuclear HMGB1 may therefore leave the genome vulnerable to DNA damage at this critical stage of cancer development. HMGB1 interacts with mismatch repair proteins MSH2 and MLH1 to perform initial damage recognition and can also mediate excision, to uphold microsatellite stability [31]. Balana and colleagues recently reported that HMGB1 can be endogenously O-GlcNAc-modified and this alters HMGB1-DNA interactions resulting in a reduced ability for DNA repair with error-prone processing of damaged DNA [32]. Mismatch repair occurs early in a subgroup of CRC and this could be a consequence of reduced HMGB1 expression. In keeping with this hypothesis, we report reduced expression of nuclear HMGB1 associated with mismatch repair protein expression within the wider CRC TMA. This is clinically relevant as microsatellite unstable tumours have a high neo-epitope load which suggests they may respond favourably to immunotherapy [33].

We show an association between HMGB1 expression intensity and density of immune cell subsets in colorectal cancer; nuclear and cytoplasmic HMBG1 associated with increased CD4^+^ lymphocytes, and nuclear HMGB1 associated with increased FOXP3^+^ and ICOS^+^ lymphocytes and reduced CD8^+^ T-cells. Meta-analyses have robustly demonstrated that CD8^+^ T-cells are associated with improved survival in CRC, and this is likely due to clonal expansion of tumour antigen-specific CD8^+^ T-cells which support an anti-tumour immune response [34,35]. Tregs can prevent the development of anti-tumour immune responses and may be associated with reduced chemosensitivity in CRC [36]. Patients with CRC have an accumulation of PD-1^+^ Tregs which impair CD8^+^ T-cell activity [37]. Our data cumulatively suggest that increased HMGB1 expression in CRC orchestrates a pro-tumour microenvironment, and HMGB1 may therefore be a therapeutic target of interest to improve endogenous anti-tumour immunity and chemosensitivity. This is in keeping with a previous study by Liu and colleagues who reported that HMGB1 knockdown in tumour cells did not impact tumour cell growth but uncovered naturally acquired long-lasting tumour specific IFN-γ or TNF-α producing CD8^+^ T-cell responses which prevented tumour cells inducing Tregs [38]

The significance of increased density of CD4^+^ T-cells and ICOS^+^ lymphocytes is less clear. Tregs commonly express CD4, and ICOS-ICOSL signalling in Tregs promotes their proliferation, survival and suppressive ability [39]. While this could fit with our hypothesis of reduced CD8^+^ T-cells and increased Tregs impacting on anti-tumour immunity, Tregs are only a small, albeit potent, sub-population of predominantly CD4^+^ T-cells in CRC. Alternatively, Th1 polarised CD4^+^ T-cells can promote anti-tumour immunity to destroy tumour cells, either directly or indirectly through effector cells such as CD8^+^ T-cells [40]. CD4^+^ lymphocytes are an incredibly diverse and plastic cellular population, and more comprehensive phenotyping of these CD4^+^ lymphocytes is important going forward to understand their impact here, such as by single-cell sequencing or flow cytometry [41]

Moving towards clinical application, we demonstrate that HMGB1 expression is associated with lymph node positivity, mismatch repair protein expression, and a distinct immune cell phenotype. These are all indicators of treatment outcomes and are used clinically to help treatment decisions [42,43]. Given the association between HMGB1 and these biomarkers, we hypothesize that HMGB1 would have utility in this clinical space. Our study was not able to assess HMGB1 expression alongside downstream treatment responses and resistance as this data is not available for this cohort. However, this would be an important next step in translational investigation. Our data also show that male patients with CRC have increased expression of cytoplasmic HMGB1, compared with female patients. This is in keeping with previous studies that have suggested sex differences, with increased HMGB1 release from stressed male cells [44,45]. Male patients have poorer outcomes from CRC and, while this is multifactorial [46], unidentified endogenous drivers of disease are likely important contributors. Exploring the association between HMGB1 expression and sex is important for future studies.

Further, ‘immune cold’ tumours within the TMA (defined by low density of CD3^+^, CD4^+^ and CD8^+^ cells) were the best prognosticator for CRC, as reported by Craig et al. [13]. Our analysis confirms that patients with ‘immune cold’ colorectal tumours have poorer overall survival rates (Supplementary File S9). We also report that strong epithelial cytoplasmic HMGB1 expression is associated with an ‘immune cold’ phenotype. Therefore, while epithelial HMGB1 expression is not directly associated with survival, it is associated with an ‘immune cold’ tumour microenvironment, which is associated with poor survival. While numbers were small, strong cytoplasmic HMGB1 was also associated with immune checkpoint markers (IDO-1, ICOS and stromal PDL1), and further investigation is needed to determine whether or not this is biologically relevant. Hubert and colleagues recently demonstrated that inhibiting HMGB1 in breast and small cell lung cancer models reduced proportions of monocytic/granulocytic myeloid-derived suppressor cells and Tregs, elicited a higher M1/M2 ratio of macrophages, and enhanced dendritic cell activation, without affecting the overall number of (CD45^+^) immune cells [47]. Such tumour microenvironment remodelling via HMGB1 blockade could work synergistically with current anti-cancer therapies, and our study suggests that a similar effect may be true for HMGB1 in CRC.

## 5. Conclusions

Overall, we reveal novel biological insight into the pathogenesis of human CRC progression. Further work is required to assess the biological consequence of HMGB1 expression in CRC to uncover novel treatment targets and biomarkers to help predict treatment responses for this malignancy.

## Figures and Tables

**Table 1:** Association between epithelial HMGB1 expression and tissue type.

Comparisons	Absent v Weak v Moderate v Strong				Absent v Weak, Moderate and Strong				Absent and Weak v Moderate and Strong				Strong v Absent, Weak and Moderate			
	Nucleus		Cytoplasm		Nucleus		Cytoplasm		Nucleus		Cytoplasm		Nucleus		Cytoplasm	
	χ^2^	*p*	χ^2^	*p*	χ^2^	*p*	χ^2^	*p*	χ^2^	*p*	χ^2^	*p*	χ^2^	*p*	χ^2^	*p*
**Colorectal Cancer TMA**																
Normal Colon vs. Colorectal Cancer	54.143	**<0.001**	36.266	**<0.001**	9.073	0.003	32.695	**<0.001**	30.747	**<0.001**	20.772	**<0.001**	51.827	**<0.001**	8.529	**0.003**
**Cancer Polyps**																
Normal vs. Adenoma	2.310	0.315	5.837	0.120	*	*	0.963	0.327	1.127	0.288	0.028	0.867	2.310	0.129	2.689	0.101
Normal vs. Carcinoma	1.346	0.510	22.491	**<0.001**	*	*	17.230	**<0.001**	1.127	0.288	16.108	**<0.001**	0.010	0.920	17.733	**<0.001**
Adenoma vs. Carcinoma	2.095	0.148	16.419	**0.001**	*	*	11.458	**0.001**	*	*	15.655	**<0.001**	2.095	0.148	9.167	**0.002**
**Non-Cancerous Polyps**																
Normal vs. Adenoma	0.280	0.868	13.484	**0.004**	*	*	7.086	**0.008**	*	*	12.066	**0.001**	0.028	0.868	4.124	**0.042**

Note: * no statistics are computed because one variable is a constant. **Bold** denotes statistically significant associations.

**Table 2:** Association between epithelial HMGB1 expression and the immune microenvironment in colonic cancer.

Comparison	Absent v Weak v Moderate v Strong				Absent v Weak, Moderate and Strong				Absent and Weak v Moderate and Strong **				Strong v Absent, Weak and Moderate			
	Nucleus		Cytoplasm		Nucleus		Cytoplasm		Nucleus		Cytoplasm		Nucleus		Cytoplasm	
	TS *	*p*	TS *	*p*	TS *	*p*	TS *	*p*	TS *	*p*	TS *	*p*	TS *	*p*	TS *	*p*
**Density (using continuous cell count data)**																
CD4+	11.134	**0.011**	15.367	**0.002**	24,128.000	**0.007**	38,520.000	**<0.001**	41,767.000	**0.007**	38,552.000	**0.013**	36,984.000	**0.010**	21,168.000	0.587
CD3+	4.710	0.194	3.472	0.324	18,399.000	0.074	34,346.000	0.358	35,565.000	0.249	33,706.000	0.444	33,358.000	0.772	19,519.000	0.268
CD8+	6.659	0.084	2.477	0.479	16,744.000	**0.022**	29,439.500	0.498	31,718.500	**0.031**	32,339.000	0.498	29,022.500	0.130	18,139.000	0.133
FOXP3+	22.489	**<0.001**	3.479	0.323	25,718.000	**0.001**	33,913.000	0.619	44,376.500	**<0.001**	34,728.000	0.873	40,366.500	**<0.001**	18,857.000	0.138
IDO-1+	3.741	0.291	6.839	0.077	21,327.000	0.186	28,967.000	0.272	34,703	0.661	30,001.000	0.077	29,730.000	0.447	16,151.000	**0.010**
ICOS+	10.026	**0.018**	10.393	**0.016**	23,326.500	**0.013**	30,417.500	0.981	39,203.500	**0.032**	29,905.000	**0.050**	35,980.500	**0.009**	16,078.000	**0.002**
CD20+	2.515	0.472	0.288	0.962	14,849.5	0.224	25,674	0.817	26,485.5	0.296	23,336.5	0.757	23,420	0.977	13,035.5	0.802
PDL1+ Stroma	2.428	0.489	8.832	**0.032**	18,957.000	0.467	27,914.000	0.064	1804.757	0.141	32,329.000	0.528	30,554.000	0.644	17,125.000	**0.027**
PDL1+ Tumour	1.364	0.714	4.839	0.184	21,178	0.353	31,450	0.797	35,824	0.897	34,860	0.409	30,942	0.817	18,594	0.259
Immune Cold vs. Immune NOS ***																
Immune Cold (low CD3/CD8)	1.972	0.578	7.446	0.059	1.895	0.169	0.560	0.454	0.864	0.353	0.108	0.742	0.352	0.553	4.841	**0.028**
Immune Cold (low CD3/CD8/CD4)	1.722	0.632	7.230	0.065	1.491	0.222	0.626	0.429	0.641	0.423	0.239	0.625	0.685	0.408	4.777	**0.029**

* TS = test statistic; Mann–Whitney U and Kruskal–Wallis tests were used for comparisons when there were two or greater than two comparator groups, respectively; ** the associations between comparator groups of ** absent + weak versus moderate + strong HMGB1 expression and immunophenotype, and *** immune cold versus immune NOS are visually represented in Figure 4. Bold text denotes significant associations, *p* < 0.05.

## Data Availability

Most data are published within this paper and within accompanying supporting files. Any additional data are available upon reasonable request to the corresponding author.

## Supplementary Materials

The following supporting information can be downloaded at: https://www.mdpi.com/article/10.3390/cancers15061865/s1, Supplementary File S1: Colorectal cancer TMA with clinico- pathological data; Supplementary File S2: Characterisation of adenomatous and cancer polyp cohorts; Supplementary File S3: Antibodies for immunohistochemistry; Supplementary File S4: Representative high power field photomicrographs of (A) weak nuclear and absent cytoplasmic (B) moderate nuclear and absent cytoplasmic (C) strong nuclear and absent cytoplasmic (D) strong nuclear and weak cytoplasmic (E) absent nuclear and moderate cytoplasmic (note blood vessel has very strong non-specific staining) and (F) strong nuclear and strong cytoplasmic staining intensities; Supplementary File S5: Association between epithelial HMGB1 expression in colonic cancer and clinico-pathological parameters; Supplementary File S6: Association between HMGB1 and overall survival in colorectal cancer; Supplementary File S7: Cancer polyp validation cohort; Supplementary File S8: Immune cell infiltrate at the invasive cancer margin of colorectal cancer polyps; Supplementary File S9: Association between immune microenvironment and overall survival in colorectal cancer.

## References

[1] Sung H., Ferlay J., Siegel R.L., Laversanne M., Soerjomataram I., Jemal A., Bray F. (2021). Global Cancer Statistics 2020: GLOBOCAN Estimates of Incidence and Mortality Worldwide for 36 Cancers in 185 Countries. CA Cancer J. Clin..

[2] Morson B.C. (1974). Evolution of cancer of the colon and rectum. Cancer.

[3] Fearon E.R., Vogelstein B. (1990). A genetic model for colorectal tumorigenesis. Cell.

[4] Guinney J., Dienstmann R., Wang X., de Reyniès A., Schlicker A., Soneson C., Marisa L., Roepman P., Nyamundanda G., Angelino P. (2015). The consensus molecular subtypes of colorectal cancer. Nat. Med..

[5] Joanito I., Wirapati P., Zhao N., Nawaz Z., Yeo G., Lee F., Eng C.L.P., Macalinao D.C., Kahraman M., Srinivasan H. (2022). Single-cell and bulk transcriptome sequencing identifies two epithelial tumor cell states and refines the consensus molecular classification of colorectal cancer. Nat. Genet..

[6] Hanahan D., Weinberg R.A. (2011). Hallmarks of cancer: The next generation. Cell.

[7] Governa V., Trella E., Mele V., Tornillo L., Amicarella F., Cremonesi E., Muraro M.G., Xu H., Droeser R., Däster S.R. (2017). The Interplay Between Neutrophils and CD8^+^ T Cells Improves Survival in Human Colorectal Cancer. Clin. Cancer Res..

[8] Chen R., Kang R., Tang D. (2022). The mechanism of HMGB1 secretion and release. Exp. Mol. Med..

[9] Kang R., Chen R., Zhang Q., Hou W., Wu S., Cao L., Huang J., Yu Y., Fan X.G., Yan Z. (2014). HMGB1 in health and disease. Mol. Asp. Med..

[10] Bertheloot D., Latz E. (2017). HMGB1, IL-1α, IL-33 and S100 proteins: Dual-function alarmins. Cell. Mol. Immunol..

[11] Sinagra T., Merlo S., Spampinato S.F., Pasquale R.D., Sortino M.A. (2015). High mobility group box 1 contributes to wound healing induced by inhibition of dipeptidylpeptidase 4 in cultured keratinocytes. Front. Pharmacol..

[12] Wu T., Zhang W., Yang G., Li H., Chen Q., Song R., Zhao L. (2016). HMGB1 overexpression as a prognostic factor for survival in cancer: A meta-analysis and systematic review. Oncotarget.

[13] Craig S.G., Humphries M.P., Alderdice M., Bingham V., Richman S.D., Loughrey M.B., Coleman H.G., Viratham-Pulsawatdi A., McCombe K., Murray G.I. (2020). Immune status is prognostic for poor survival in colorectal cancer patients and is associated with tumour hypoxia. Br. J. Cancer.

[14] Porter R.J., Murray G.I., Alnabulsi A., Humphries M.P., James J.A., Salto-Tellez M., Craig S.G., Wang J.M., Yoshimura T., McLean M.H. (2021). Colonic epithelial cathelicidin (LL-37) expression intensity is associated with progression of colorectal cancer and presence of CD8^+^ T cell infiltrate. J. Pathol. Clin. Res..

[15] Alnabulsi A., Swan R., Cash B., Alnabulsi A., Murray G.I. (2017). The differential expression of omega-3 and omega-6 fatty acid metabolising enzymes in colorectal cancer and its prognostic significance. Br. J. Cancer.

[16] Porter R.J., Murray G.I., Brice D.P., Petty R.D., McLean M.H. (2020). Novel biomarkers for risk stratification of Barrett’s oesophagus associated neoplastic progression-epithelial HMGB1 expression and stromal lymphocytic phenotype. Br. J. Cancer.

[17] McLean M.H., Murray G.I., Stewart K.N., Norrie G., Mayer C., Hold G.L., Thomson J., Fyfe N., Hope M., Mowat N.A. (2011). The inflammatory microenvironment in colorectal neoplasia. PLoS ONE.

[18] Bankhead P., Loughrey M.B., Fernández J.A., Dombrowski Y., McArt D.G., Dunne P.D., McQuaid S., Gray R.T., Murray L.J., Coleman H.G. (2017). QuPath: Open source software for digital pathology image analysis. Sci. Rep..

[19] Scottish Bowel Screening Programme Statistics: For the Period of Invitations from May 2019 to April 2021. National Statistics Publication. Public Health Scotland. 8 February 2022. https://publichealthscotland.scot/media/11605/2022-02-08-bowel-screening-publication-report.pdf.

[20] Bindea G., Mlecnik B., Galon J. (2022). Immune sunrise: From the immunome to the cancer immune landscape. Oncoimmunology.

[21] Yalcin S., Philip P.A., Athanasiadis I., Bazarbashi S., Shamseddine A. (2022). Classification of early-stage colon cancer with Immunoscore^®^: Clinical evidence and case studies. Future Oncol..

[22] Li Z., Wang H., Song B., Sun Y., Han J., Xu Z. (2015). Expression of high mobility group box-1 in colorectal cancer and its clinical significance. Zhonghua Wei Chang Wai Ke Za Zhi.

[23] Peng R.Q., Wu X.J., Ding Y., Li C.Y., Yu X.J., Zhang X., Pan Z.Z., Wan D.S., Zheng L.M., Zeng Y.X. (2010). Co-expression of nuclear and cytoplasmic HMGB1 is inversely associated with infiltration of CD45RO+ T cells and prognosis in patients with stage IIIB colon cancer. BMC Cancer.

[24] Wang C.Q., Huang B.F., Wang Y., Tang C.H., Jin H.C., Shao F., Shao J.K., Wang Q., Zeng Y. (2020). Subcellular localization of HMGB1 in colorectal cancer impacts on tumor grade and survival prognosis. Sci. Rep..

[25] Kim J.C., Bodmer W.F. (2022). Genomic landscape of colorectal carcinogenesis. J. Cancer Res. Clin. Oncol..

[26] Rowell J.P., Simpson K.L., Stott K., Watson M., Thomas J.O. (2012). HMGB1-facilitated p53 DNA binding occurs via HMG-Box/p53 transactivation domain interaction, regulated by the acidic tail. Structure.

[27] Yan H.X., Wu H.P., Zhang H.L., Ashton C., Tong C., Wu H., Qian Q.J., Wang H.Y., Ying Q.L. (2013). p53 promotes inflammation-associated hepatocarcinogenesis by inducing HMGB1 release. J. Hepatol..

[28] Su Z., Lu H., Jiang H., Zhu H., Li Z., Zhang P., Ni P., Shen H., Xu W., Xu H. (2015). IFN-γ-producing Th17 cells bias by HMGB1-T-bet/RUNX3 axis might contribute to progression of coronary artery atherosclerosis. Atherosclerosis.

[29] Davalos A.R., Kawahara M., Malhotra G.K., Schaum N., Huang J., Ved U., Beausejour C.M., Coppe J.P., Rodier F., Campisi J. (2013). p53-dependent release of Alarmin HMGB1 is a central mediator of senescent phenotypes. J. Cell Biol..

[30] Taverna S., Tonacci A., Ferraro M., Cammarata G., Cuttitta G., Bucchieri S., Pace E., Gangemi S. (2022). High Mobility Group Box 1: Biological Functions and Relevance in Oxidative Stress Related Chronic Diseases. Cells.

[31] Yuan F., Gu L., Guo S., Wang C., Li G.M. (2004). Evidence for involvement of HMGB1 protein in human DNA mismatch repair. J. Biol. Chem..

[32] Balana A.T., Mukherjee A., Nagpal H., Moon S.P., Fierz B., Vasquez K.M., Pratt M.R. (2021). O-GlcNAcylation of High Mobility Group Box 1 (HMGB1) Alters Its DNA Binding and DNA Damage Processing Activities. J. Am. Chem. Soc..

[33] Din S., Wong K., Mueller M.F., Oniscu A., Hewinson J., Black C.J., Miller M.L., Jiménez-Sánchez A., Rabbie R., Rashid M. (2018). Mutational Analysis Identifies Therapeutic Biomarkers in Inflammatory Bowel Disease-Associated Colorectal Cancers. Clin. Cancer Res..

[34] Idos G.E., Kwok J., Bonthala N., Kysh L., Gruber S.B., Qu C. (2020). The Prognostic Implications of Tumor Infiltrating Lymphocytes in Colorectal Cancer: A Systematic Review and Meta-Analysis. Sci. Rep..

[35] Alexander P.G., McMillan D.C., Park J.H. (2020). The local inflammatory response in colorectal cancer—Type, location or density? A systematic review and meta-analysis. Cancer Treat. Rev..

[36] Ning T., Li J., He Y., Zhang H., Wang X., Deng T., Liu R., Li H., Bai M., Fan Q. (2021). Exosomal miR-208b related with oxaliplatin resistance promotes Treg expansion in colorectal cancer. Mol. Ther..

[37] Fujimoto H., Saito Y., Ohuchida K., Kawakami E., Fujiki S., Watanabe T., Ono R., Kaneko A., Takagi S., Najima Y. (2018). Deregulated Mucosal Immune Surveillance through Gut-Associated Regulatory T Cells and PD-1^+^ T Cells in Human Colorectal Cancer. J. Immunol..

[38] Liu Z., Falo L.D., You Z. (2011). Knockdown of HMGB1 in tumor cells attenuates their ability to induce regulatory T cells and uncovers naturally acquired CD8 T cell-dependent antitumor immunity. J. Immunol..

[39] Li D.Y., Xiong X.Z. (2020). ICOS^+^ Tregs: A Functional Subset of Tregs in Immune Diseases. Front. Immunol..

[40] Galaine J., Turco C., Vauchy C., Royer B., Mercier-Letondal P., Queiroz L., Loyon R., Mouget V., Boidot R., Laheurte C. (2019). CD4 T cells target colorectal cancer antigens upregulated by oxaliplatin. Int. J. Cancer.

[41] DiToro D., Basu R. (2021). Emerging Complexity in CD4^+^T Lineage Programming and Its Implications in Colorectal Cancer. Front. Immunol..

[42] Bromham N., Kallioinen M., Hoskin P., Davies R.J. (2020). Colorectal cancer: Summary of NICE guidance. BMJ.

[43] André T., Shiu K.K., Kim T.W., Jensen B.V., Jensen L.H., Punt C., Smith D., Garcia-Carbonero R., Benavides M., Gibbs P. (2020). KEYNOTE-177 Investigators. Pembrolizumab in Microsatellite-Instability-High Advanced Colorectal Cancer. N. Engl. J. Med..

[44] Zemskova M., Kurdykov S., James J., McClain N., Rafikov R., Rafikov O. (2020). Sex-specific stress response and HMGB1 release in pulmonary endothelial cells. PLoS ONE.

[45] Mohamed R., Rafikova O., O’Connor P.M., Sullivan J.C. (2020). Greater high-mobility group box 1 in male compared with female spontaneously hypertensive rats worsens renal ischemia-reperfusion injury. Clin. Sci..

[46] White A., Ironmonger L., Steele R.J.C., Ormiston-Smith N., Crawford C., Seims A. (2018). A review of sex-related differences in colorectal cancer incidence, screening uptake, routes to diagnosis, cancer stage and survival in the UK. BMC Cancer.

[47] Hubert P., Roncarati P., Demoulin S., Pilard C., Ancion M., Reynders C., Lerho T., Bruyere D., Lebeau A., Radermecker C. (2021). Extracellular HMGB1 blockade inhibits tumor growth through profoundly remodeling immune microenvironment and enhances checkpoint inhibitor-based immunotherapy. J. Immunother. Cancer.

