# Supplementary material for: Subcellular Epithelial HMGB1 Expression Is Associated with Colorectal Neoplastic Progression, Male Sex, Mismatch Repair Protein Expression, Lymph Node Positivity, and an ‘Immune Cold’ Phenotype Associated with Poor Survival
Source: Cancers (Basel). 2023 Mar 20;15(6):1865. doi: 10.3390/cancers15061865 (PMC10047220; doi:10.3390/cancers15061865)
Supplement: Supplementary file 1 [file cancers-15-01865-s001.zip › cancers-2005442-supplementary.pdf]

SUPPLEMENTARY FILES - MATERIALS, TABLES AND DATA

Supplementary File S1 – page 2
Table: Colorectal cancer TMA with clinico- pathological data

Supplementary File S2 – page 3
Characterisation of adenomatous and cancer polyp cohorts

Supplementary File S3 – page 5
Table: Antibodies for immunohistochemistry

Supplementary File S4 – page 6
Representative high power field photomicrographs of different HMGB1 epithelial nuclear and cytoplastic staining intensities.

Supplementary File S5 – page 7
Table: Association between epithelial HMGB1 expression in colonic cancer and clinico-pathological parameters.

Supplementary File S6 – page 8
Association between HMGB1 and overall survival in colorectal cancer

Supplementary File S7 – page 10
Cancer polyp validation cohort with molecular data

Supplementary File S8 – page 12
Immune cell infiltrate at the invasive cancer margin of colorectal cancer polyps

Supplementary File S9 – page 13
Association between immune microenvironment and overall survival in colorectal cancer

Supplementary File S1 – Table: Colorectal cancer TMA with clinico- pathological data

Characteristic	No patients	Percentage	Relationship with survival
**Sex**			
Male	340	52.3	χ2= 0.027, p=0.870
Female	310	47.7	
**Age**			
<70	305	46.9	χ2=29.213, **p<0.001**
≥70	345	53.1	
**Screening Detected**			
Yes	52	8	χ2=16.381, **p<0.001**
No	598	92	
**Tumour Site**			
Proximal colon	261	40.2	Proximal v distal, χ2= 8.418, **p=0.004**
Distal colon	245	37.7	Distal v rectal, χ2= 0.906, p=0.341
Rectum	144	22.2	Colon v rectum, χ2=0.098, p=0.754
**Tumour Differentiation**			
Well/Moderate	600	92.3	χ2=0.976, p=0.323
Poor	50	7.7	
**Extra-Mural Venous Invasion**			
Present	140	21.5	χ2=100.946, **p<0.001**
Absent	510	78.5	
**Mismatch Repair Protein Status (defined by MLH1 and MSH2 status)**			
Deficient	96	15.2	χ2=2.848, p=0.091
Proficient	536	84.8	
**pT Stage**			
T1	30	4.6	T1 v T2, χ2=0.382, p=0.536
T2	114	17.5	T2 v T3, χ2=24.739, **p<0.001**
T3	411	63.2	T3 v T4, χ2=30.159, **p<0.001**
T4	95	14.6	
**pN Stage**			
N0	364	56	N0 v N1, χ2=54.071, **p<0.001**
N1	177	27.2	N1 v N2, χ2=17.636, **p<0.001**
N2	109	16.8	
**Dukes Stage**			
A	120	18.5	A v B, χ2=5.059, **p=0.025**
B	244	37.5	B v C, χ2=65.510, **p<0.001**
C	286	44	

Supplementary File S2 – Characterisation of adenomatous and cancer polyp cohorts

Adenomatous Polyps

Clinico-pathological data were available for 52/52 (100%) adenomatous polyps. Non-cancerous adenomatous polyps were retrieved from 19 (37%) female and 33 (63%) male patients, with a median age of 61 (75, 68) years. Site was from the colon in 43 (82%), rectum in 5 (10%), and rectosigmoid junction in 4 (8%) patients. Of colonic adenomas, 26 (60%) were from the sigmoid, 7 (17%) were from the ascending colon, 6 (14%) were from the transverse colon, 3 (7%) were from the descending colon, and 1 (2%) was not accurately reported. Histology reported 31 (60%) as tubular, 14 (27%) as tubulovillous, 5 (9%) as serrated or sessile, and 2 (4%) were not reported accurately. 46 (88%) had low grade and 6 (12%) had high grade dysplasia. The median size was 9.5 (6.3, 12) mm, at the largest measurement. 17 (33%) patients with polyps were identified by the NHS Scotland Bowel Screening Program, whereas this was not true (or not documented) for 35 (67%) patients.

Initial Cancer Polyp (CaP) Cohort

Clinico-pathological data were available for 24/28 (86%) colorectal cancer polyps (CaP). CaP were retrieved from 8 (33%) female and 16 (67%) male patients, with a median age of 72.5 (70.25, 75) years. Polyp site was unknown for 3 (12%), from the colon in 15 (63%), and from the rectum in 6 (25%) patients. Of colonic CaP, 11 (73%) were from the sigmoid, 2 (12%) were from the transverse colon, 1 (7%) was from the descending colon, and 1 (7%) was unspecified. Background polyp histology reported 9 (38%) as tubular, 2 (8%) as tubulovillous, 2 (8%) as sessile, 1 (4%) as sessile tubular, 1 (4%) as ulcerated and 9 (38%) as unknown. Dysplasia within the CaP was reported as 'high' for 9 (38%), 'low' for 4 (17%), 'mostly low with foci of high' for 5 (20%), 'moderate' for 2 (8%), or unknown for 4 (17%). The median CaP size was 12 (10.5, 16) mm, at the largest measurement. 11 (46%) patients with CaP were identified via the NHS Scotland Bowel Screening Program, whereas this was not true (or not documented) for 13 (54%) patients. 3 patients were noted to have diverticulosis; no patients were reported to have inflammatory bowel diseases or genetic colorectal cancer syndromes such as Familial Adenomatous Polyposis or Hereditary Non-Polyposis Colorectal Cancer.

Validation Cancer Polyp (CaP) Cohort

Aberrant HMGB1 expression was observed at the invasive cancer margin in our initial CaP cohort (n=28). We wanted to confirm this in a validation cohort, and investigate whether HMGB1 expression was associated with molecular markers.

41 CaP lesions were obtained from 33/41 (80.5%) patients *via* endoscopic polypectomy, 4/41 (9.8%) patients *via* endoscopic mucosal resection polypectomy, and 4/41 (9.8%) patients *via* transanal minimally invasive surgery polypectomy. 17/41 (41.5%) were detected through the NHS Scotland Bowel Cancer Screening Programme.

CaPs were removed from the rectum in 13 (31.7%) patients, recto-sigmoid junction in 2 (4.9%) patients, sigmoid colon in 21 (51.2%) patients, descending colon in 3 (7.3%) patients, splenic flexure in 1 (2.4%) patient, and ascending colon in 1 (2.4%) patient. Polyp size was <10mm for 1 (2.4%) patient, 10-20mm for 29 (70.7%) patients, ≥ 20mm for 10 (24.4%) patients, and

unknown for 1 (2.4%) patient. While only one CaP was identified and included for analysis, some patients had synchronous polyps resected. The number of polyps resected per patient was unknown (‘multiple mucosal fragments’) for 1 (2.4%) patient, one for 36 (87.8%) patients, two for 2 (4.9%) patients, three for 1 (2.4%) patient, and four for 1 (2.4%) patient. Background adenomatous tissue in CaP was reported as tubular for 11 (26.8%) CaP, tubulo-villous for 11 (26.8%) CaP, sessile serrated for 1 (2.4%) CaP, and unspecified for 18 (43.9%) CaP. All foci of cancer were adenocarcinoma.

KRAS, BRAF, EGFR and MSI status were available for most tumours. MSI status was not tested in 17 (41.5%) CaP, proficient in 21 (51.2%) CaP, deficient in 1 (2.4%) CaP, and inconclusive/poor quality in 2 (4.9%) CaP. *KRAS* status was not tested in 16 (39%) CaP, wildtype in 11 (26.8%) CaP, mutated in 13 (31.7%) CaP, or inconclusive/poor quality in 2 (4.9%) CaP. Specifically, 3 (23%) *KRAS* mutations were c.35G>A; p.(Gly12Asp), 3 (23%) were c.35G>T;p.(Gly12Val), 3 (23%) were c.38G>A;p.(Gly13Asp), 1 (8%) was c.34G>T;p.Gly12Cys, 1 (8%) was c.182A>G; p.(Gln61Arg), 1 (8%) was c.348_349insG; p.(Lys117Glufs*3), and 1 (8%) was mutation in codon 12. *BRAF* status was not tested in 17 (41.5%) CaP, not mutated in 23 (56.1%) CaP, and inconclusive or poor quality in 1 (2.4%) CaP. *EGFR* status was not tested in 35 (85.4%) CaP, not expressed 1 (2.4%) CaP, expressed in 4 (9.8%) CaP, and inconclusive or poor quality in 1 (2.4%) CaP.

Supplementary File S3 – Table: Antibodies for immunohistochemistry

Antibody target	Antibody type	Antigen retrieval	Dilution	Incubation time (min)	Positive control	Supplier	Code	Isotype, clone
HMGB1	rabbit monoclonal	citrate	1:400	60	colorectal cancer	abcam	ab79823	IgG, EPR3507
p53	mouse monoclonal	EDTA	1:250	60	Barrett's oesophagus with dysplasia	abcam	ab1101	IgG2a, DO-1
RUNX3	mouse monoclonal	EDTA	1:500	60	colorectal cancer	abcam	ab40278	IgG1, R3-5G4
CD20+ B-cells	mouse monoclonal	citrate	1:600	60	tonsil	Agilent	M 075529-2	IgG2a, L26
CD4+ T-cells	mouse monoclonal	EDTA	1:500	60	tonsil	abcam	ab133616	IgG, EPR6855
CD8+ T-cells	mouse monoclonal	EDTA	1:150	60	tonsil	abcam	ab17147	IgG1, 144B
FOXP3+ T-cells	mouse monoclonal	EDTA	1:200	60	tonsil	abcam	ab20034	IgG1, 236A/E7
CD68+ macrophages	mouse monoclonal	EDTA	1:100	60	tonsil	abcam	ab955	IgG1, KP1
Antibodies used for assessment of lymphocyte infiltrate and immune checkpoint biomarkers in the colorectal cancer TMA from[17]								
CD3+ T-cells	rabbit monoclonal	CC1	Neat	32	tonsil	Ventana	790-4341	IgG, 2GV6
CD4+ T-cells	rabbit monoclonal	CC1	Neat	60	tonsil	Ventana	790-4423	IgG, SP35
CD8+ T-cells	mouse monoclonal	ER2	1:50	20	tonsil	Dako	M7103	IgG1, CD/144B
FoxP3+ T-cells	rabbit monoclonal	CC1	1:50	48	tonsil	LSBio	LS-C210349	IgG, SP97
CD20+ B-cells	mouse monoclonal	ER1	1:400	30	tonsil	Dako	M0755	IgG2a, L26
IDO-1	rabbit monoclonal	ER2	1:400	20	tonsil	Cell Signalling	#86630	IgG, D5J4E
ICOS	rabbit monoclonal	ER2	1:400	20	tonsil	Cell Signalling	#89601	IgG, D1K2T
PDL-1	rabbit monoclonal	CC1	Neat	64	tonsil	Ventana	790-4905	IgG, SP263

Supplementary File S4. Representative high power field photomicrographs of (A) weak nuclear and absent cytoplasmic (B) moderate nuclear and absent cytoplasmic (C) strong nuclear and absent cytoplasmic (D) strong nuclear and weak cytoplasmic (E) absent nuclear and moderate cytoplasmic (note blood vessel has very strong non-specific staining) and (F) strong nuclear and strong cytoplasmic staining intensities.

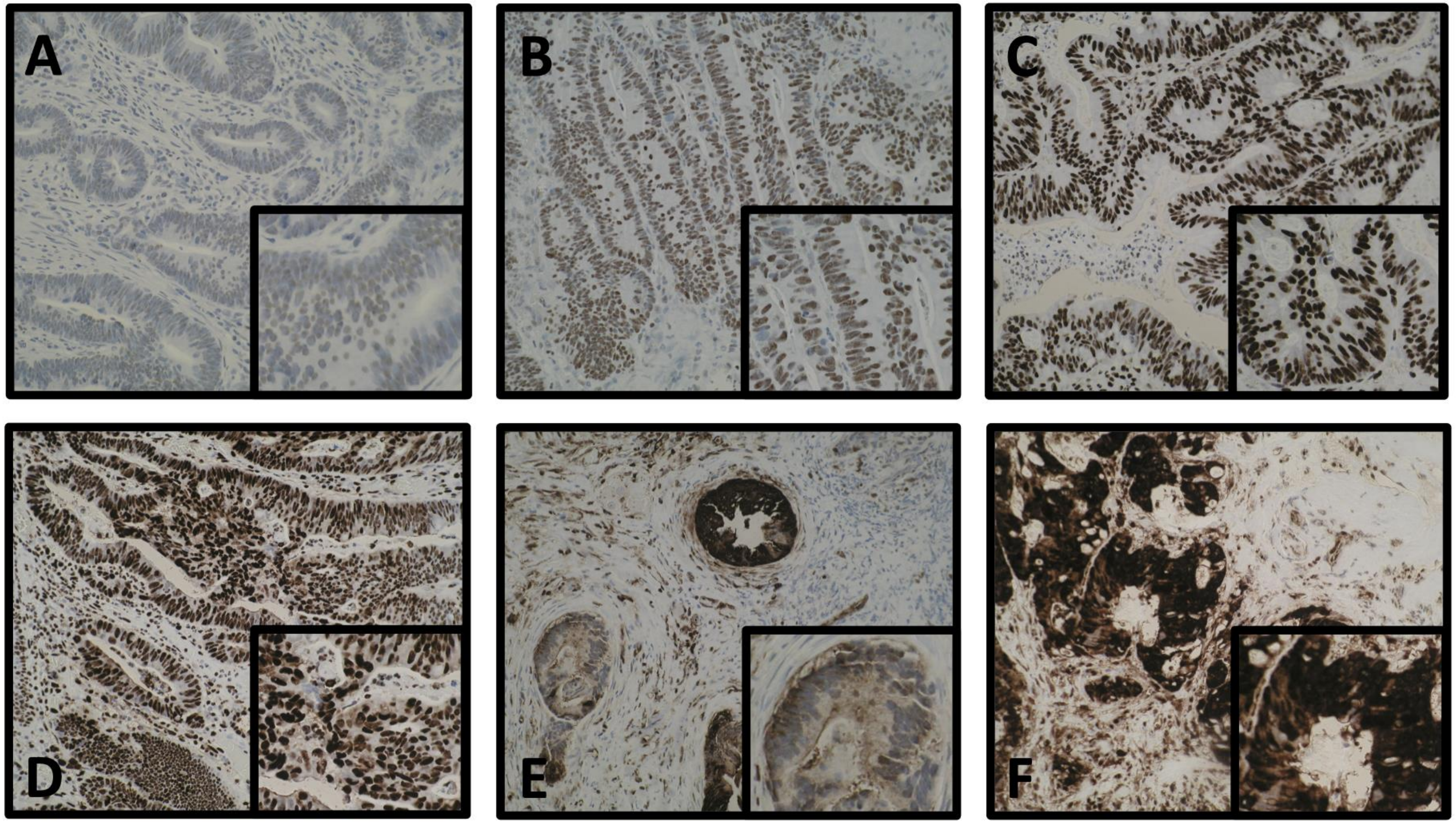

Supplementary File S5 – Table: Association between epithelial HMGB1 expression in colonic cancer and clinico-pathological parameters

Clinico-Pathological Parameter	Absent v weak v moderate v strong				Absent v weak, moderate and strong				Absent and weak v moderate and strong				Strong v absent, weak and moderate			
	Nucleus		Cytoplasm		Nucleus		Cytoplasm		Nucleus		Cytoplasm		Nucleus		Cytoplasm	
	**χ2**	**p**	**χ2**	**p**	**χ2**	**p**	**χ2**	**p**	**χ2**	**p**	**χ2**	**p**	**χ2**	**p**	**χ2**	**p**
Sex	1.665	0.645	9.796	**0.020**	0.229	0.632	5.885	**0.015**	0.696	0.404	6.785	**0.009**	0.092	0.762	6.785	**0.009**
Age at Surgery (Banded <70, ≥70)	1.699	0.637	11.281	**0.010**	0.644	0.415	3.631	0.057	0.209	0.647	0.058	0.810	1.190	0.275	0.058	0.810
Screening Detected	1.251	0.741	2.770	0.428	0.206	0.650	1.216	0.270	0.000	0.992	2.134	0.144	0.631	0.427	2.134	0.144
Tumour Site (colon v rectum)	3.532	0.317	0.642	0.887	3.391	0.066	0.149	0.699	2.572	0.210	0.615	0.433	0.503	0.478	0.615	0.433
Tumour Site (distal, proximal, rectum)	7.317	0.293	1.988	0.921	7.028	**0.030**	0.621	0.733	2.541	0.281	0.630	0.730	1.296	0.523	0.630	0.730
Tumour Differentiation (poor, mod, well)	6.243	0.396	8.576	0.199	3.431	0.180	4.332	0.115	4.145	0.126	4.686	0.096	0.212	0.899	4.686	0.096
Tumour Differentiation (poor v well/mod)	5.547	0.136	5.460	0.141	3.414	0.065	2.708	0.100	3.801	0.051	2.299	0.129	0.201	0.654	2.299	0.129
EMVI	3.572	0.312	3.251	0.355	0.266	0.606	0.336	0.562	0.768	0.381	2.884	0.089	2.334	0.127	2.884	0.089
Mismatch Repair	11.250	**0.010**	0.854	0.837	10.119	**0.001**	0.007	0.933	0.485	0.486	0.613	0.434	0.248	0.619	0.613	0.434
TNM Stage	59.570	**0.008**	50.336	0.057	14.838	0.250	15.949	0.194	14.319	0.281	13.267	0.350	21.495	**0.044**	13.267	0.350
T Stage	23.272	**0.006**	8.355	0.499	10.838	**0.013**	3.770	0.287	5.307	0.151	2.603	0.457	2.608	0.456	2.603	0.457
N Stage	10.862	0.093	9.934	0.127	3.462	0.177	3.665	0.160	0.790	0.674	3.530	0.171	3.094	0.213	3.530	0.171
Lymph Nodes Positive	47.932	0.596	69.919	**0.040**	17.860	0.398	15.569	0.555	13.422	0.708	40.627	**<0.001**	11.023	0.855	40.627	**<0.001**
Dukes' Stage	15.551	**0.016**	8.686	0.192	5.611	0.600	7.764	**0.021**	1.438	0.487	0.789	0.674	3.526	0.172	0.789	0.674

Supplementary File S6 - Association between HMGB1 and overall survival in colorectal cancer

Kaplan Meier Survival Analysis for A) nuclear HMGB1 and B) cytoplasmic HMGB1 in the colorectal tissue microarray.

A) Association between nuclear HMGB1 expression and survival, in colorectal cancer

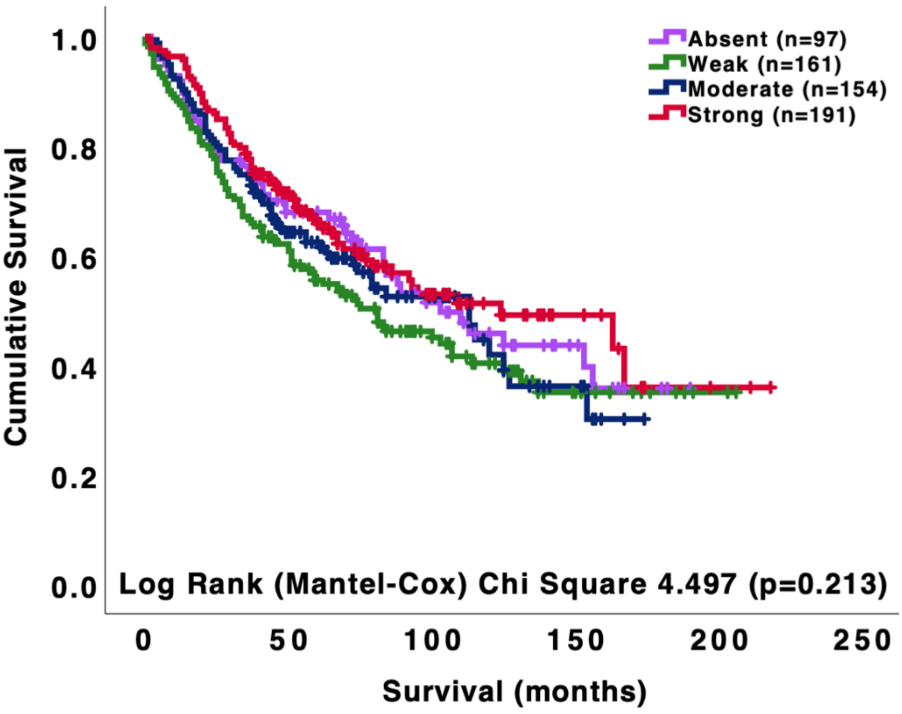

No. at risk

**Absent**	**97**	**61**	**29**	**12**	**0**	**0**
**Weak**	**161**	**95**	**42**	**14**	**0**	**0**
**Moderate**	**154**	**77**	**29**	**8**	**0**	**0**
**Strong**	**191**	**114**	**38**	**11**	**2**	**0**

B) Association between cytoplasmic HMGB1 expression and survival, in colorectal cancer

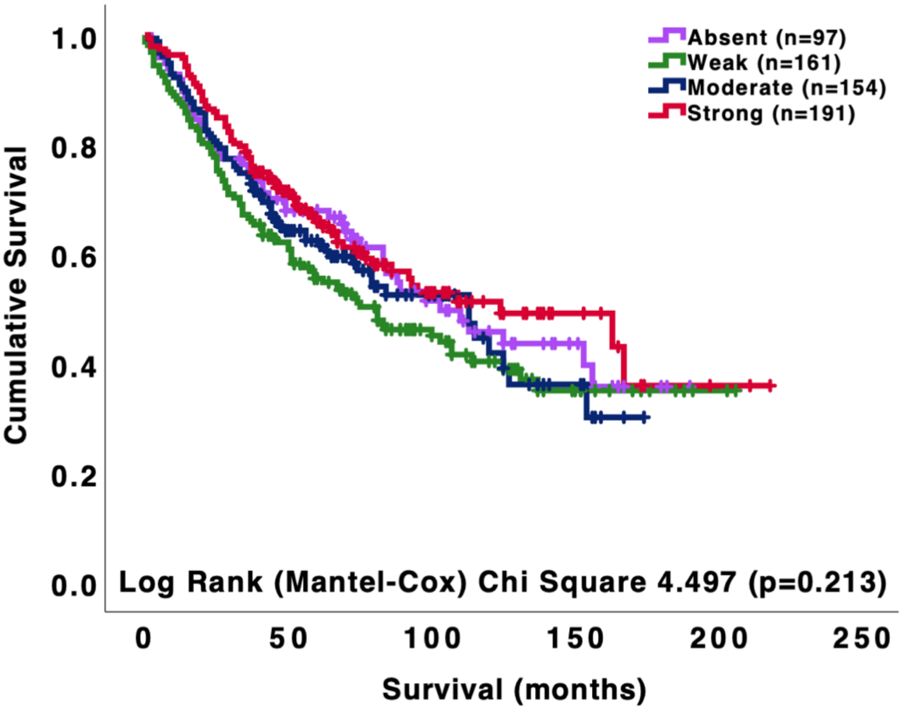

No. at risk

**Absent**	**97**	**61**	**29**	**12**	**0**	**0**
**Weak**	**161**	**95**	**42**	**14**	**0**	**0**
**Moderate**	**154**	**77**	**29**	**8**	**0**	**0**
**Strong**	**191**	**114**	**38**	**11**	**2**	**0**

Supplementary File S7 - Cancer polyp validation cohort

The CaP validation cohort **(B)** demonstrates the same expression pattern of HMGB1 as the initial CaP discovery cohort **(A)**.

A) Discovery / Initial Cohort

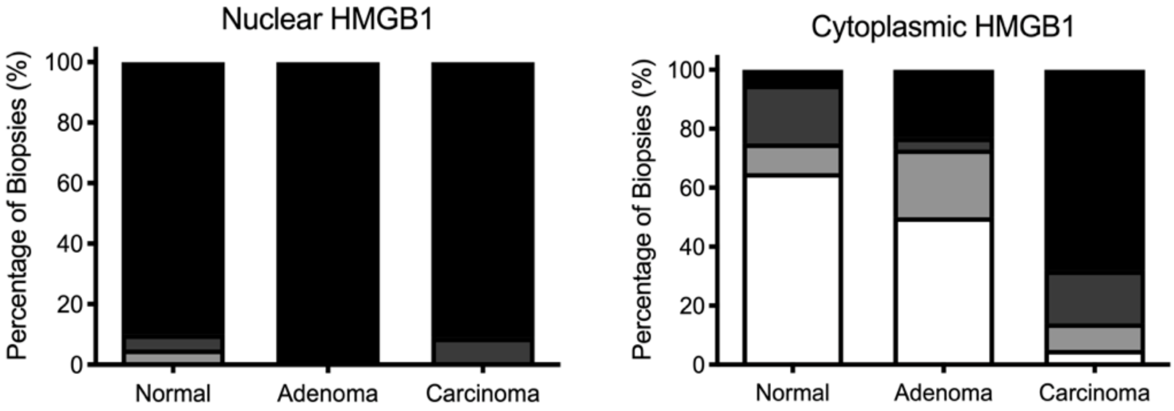

B) Validation Cohort

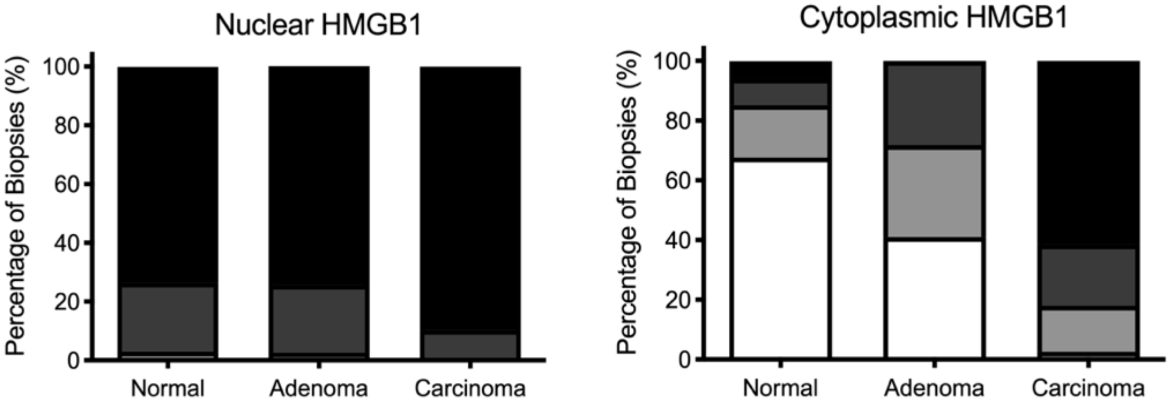

Comparisons	Absent v weak v moderate v strong				Absent v weak, moderate and strong				Absent and weak v moderate and strong				Strong v absent, weak and moderate			
	Nucleus		Cytoplasm		Nucleus		Cytoplasm		Nucleus		Cytoplasm		Nucleus		Cytoplasm	
	χ2	p	χ2	p	χ2	p	χ2	p	χ2	p	χ2	p	χ2	p	χ2	p
**Cancer Polyps – Initial Cohort**																
Normal v Adenoma	2.310	0.315	5.837	0.120	*	*	0.963	0.327	1.127	0.288	0.028	0.867	2.310	0.129	2.689	0.101
Normal v Carcinoma	1.346	0.510	22.491	**<0.001**	*	*	17.230	**<0.001**	1.127	0.288	16.108	**<0.001**	0.010	0.920	17.733	**<0.001**
Adenoma v Carcinoma	2.095	0.148	16.419	**0.001**	*	*	11.458	**0.001**	*	*	15.655	**<0.001**	2.095	0.148	9.167	**0.002**
**Cancer Polyps – Validation Cohort**																
Normal v Adenoma	0.013	1.000	9.530	**0.023**	*	*	5.174	**0.023**	0.010	0.922	1.934	0.257	0.006	0.936	2.359	0.125
Normal v Carcinoma	3.675	0.159	40.904	**<0.001**	*	*	34.865	**<0.001**	1.163	0.281	32.959	**<0.001**	3.263	0.071	24.537	**<0.001**
Adenoma v Carcinoma	3.486	0.175	39.709	**<0.001**	*	*	16.924	**<0.001**	1.013	1.000	22.856	**<0.001**	3.134	0.138	34.667	**<0.001**

Note. *no statistics are computed because one variable is a constant.

Supplementary File S8 - Immune cell infiltrate at the invasive cancer margin of colorectal cancer polyps

Immune cell population	Number of CaP	Median number of positive cells (SEM)	25th percentile	75th percentile
$CD20^+$ B-cells	21	46.52 (18.72)	6	35
$CD4^+$ T-cells	21	215.38 (31.55)	114	312
$CD8^+$ T-cells	21	89.67 (13.58)	44	114
$FOXP3^+$ Tregs	21	69.14 (11.06)	25	110
$CD68^+$ macrophages	19	231.26 (34.03)	106.5	328.5

Supplementary File S9 - Association between immune microenvironment and overall survival in colorectal cancer

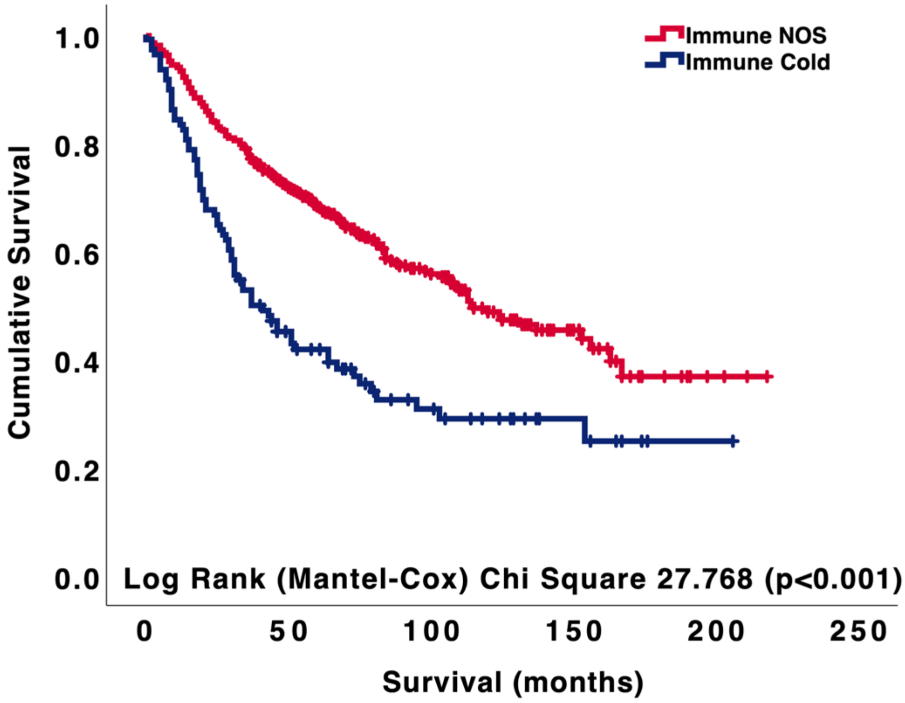

No. at risk

**Immune Cold**	**108**	**41**	**18**	**7**	**1**	**0**
**Immune NOS**	**444**	**277**	**108**	**32**	**3**	**0**
